# Global health research case studies: lessons from partnerships addressing health inequities

**DOI:** 10.1186/1472-698X-11-S2-S2

**Published:** 2011-11-08

**Authors:** Zoë Boutilier, Ibrahim Daibes, Erica Di Ruggiero

**Affiliations:** 1 Global Health Research Initiative, 150 Kent Street, Ottawa, Canada; 2 Institute of Population and Public Health, Canadian Institutes of Health Research, 600 Peter Morand Crescent, Suite 312, Ottawa, Canada

## 

Inspiration for compiling this collection of case studies comes from the Global Health Research Initiative’s (GHRI) commitment to conceptualizing and supporting global health research as a practice with increasingly discernable core characteristics. Through an exploration of these characteristics, the collection highlights practical, relevant and transferable lessons for consideration by researchers, their research-user partners, and donors working to address health inequities through global health research partnerships. The value of global health research partnerships is illustrated through the achievements of the collaborations featured in this collection.

The ten case studies included in this collection do not describe individual research projects. Instead, they each provide an in-depth account of a defined program of research that acts as a platform for theoretically linked research projects. The programs are an integrated blend of knowledge generation, capacity building, and knowledge translation activities that have evolved towards increasing complexity and sophistication. In particular, attention to capacity building and knowledge translation increases as the programs mature over time. The programs of research are animated by a core alliance of individuals whose international partnerships are rooted in mutual trust and the articulation of a common goal: health equity.

The cases presented in this collection are concerned with health inequities experienced by certain population groups. For example, the two cases set in South Asia (Haddad et al., Mumtaz et al.) are both concerned with the persistent health inequities that are experienced by lower-caste women belonging to marginalized indigenous groups. Another disadvantaged population group highlighted twice in this collection is people living with HIV/AIDS in rural Sub-Saharan Africa (Kipp et al., Sodhi et al.). A third group, Ecuadorians with limited resources who are vulnerable to environmental degradation and to acute pesticide poisoning, is also highlighted twice in this collection (Spiegel et al., Cole et al.). All of these groups face persistent social and health inequities that have “both historical roots and present day causes” (Cole et al.).

This collection features partnerships that include Canadian researchers. This is in part not accidental given that these cases were compiled by Canada’s Global Health Research Initiative (GHRI), a partnership between five Canadian government agencies that are responsible for health, health research and international development (the International Development Research Centre, the Canadian Institutes of Health Research, Health Canada, the Canadian International Development Agency, and the Public Health Agency of Canada). Over the past ten years, GHRI has sought to understand the characteristics of effective global health research and to create an environment that is conducive to its successful conduct. While the programs described in this collection are not all directly supported by GHRI, they share characteristics that are common to the programs of research supported by GHRI. We emphasize these characteristics here because we believe that they are core to the practice of global health research. The practice of global health research as described in these case studies and as supported by GHRI is characterized by:

1) long-term and sustainable North-South partnerships;

2) interdisciplinary responses to complex issues;

3) participatory action research that grounds the research in its context; and

4) research with a policy or practice impact orientation.

In this introductory essay we elaborate on each of these characteristics. We also take this opportunity to highlight some of the commendable achievements of the partnerships. At the same time, we do not neglect to expand on the challenges that face global health research partnerships, nor fail to recognize the systemic barriers that too often confine researchers, research-users, and donors.

## Long-term, sustainable North-South partnerships

The complexity of health issues addressed by global health research programs necessitates long-term visions and timelines. On average, the partnerships described herein have been in existence for just short of a decade; in two cases the partnership has been in existence for almost a decade and a half (Haddad et al., Kipp et al.). This commitment to long-term, North-South partnerships is significant given that political, institutional, and professional priorities tend to change with time. Literature on North-South partnerships is often pessimistic about the prospects for partnership sustainability, with repeated references to pervasive power imbalances in agenda-setting, in funding sources, and in allegiance to methodologies and scientific traditions [1-3]. These and other issues present an ongoing challenge to attempts to establish and maintain long-term North-South research partnerships. Despite these challenges, the case studies in this collection demonstrate that partnerships of this kind can not only be sustained, but can thrive.

The sustainability of these partnerships might be a product – or a cause – of a continued evolution in sophistication and approach. In the case of Delisle et al., the process of exploring the initial research questions and assumptions led to new ones that needed to be tested. The generation of scientific knowledge, often the initial impetus for the partnership, was enhanced over time by an increasing investment in capacity building and knowledge translation activities. In the words of Haddad et al., “The initial focus on survey-based research and data analysis gradually transformed in the direction of understanding local governance, political analysis, marginalization, gender and empowerment” [4]. In most of the cases, the overall program of research systematically emerged from its component parts. For example, Cole et al. describe their progression through three distinctly-funded projects (‘EcoHealth II’ funded from 2005 to 2008, ‘Healthy Horticulture’ funded from 2007 to 2010, and ‘Social Capital and Accountability’ funded from 2008 to 2011). Each project was designed to build on the last; not just in terms of the scientific knowledge generated, but also in terms of the human capacity developed and the impact on policy and practice. Similarly, Spiegel et al. describe the phases that made up their Ecuador EcoHealth program: a nationally-accredited ‘train-the-trainers’ Master’s program led to the establishment of other Masters programs and eventually to the launch of an innovative doctoral program. The evolution of the research program seems to reflect an evolving understanding of the problem, a greater appreciation for nuances and context, and the consolidation of the numerous relationships that must be in place for the purposes of credibility.

Another key similarity that links these longstanding, multi-stage, and evolving programs of research is their success in attracting funds from different sources over time. This may seem obvious, given that many of these partnerships have been in existence for almost a decade and given that donors generally do not commit to ten year timelines. It is instructive nonetheless to observe that these programs of research were sufficiently multi-faceted and compelling to be supported by a series of different donors, each with unique (albeit sometimes overlapping) mandates. The research program of Yassi et al. is one good example, having received at various times support from sources including (but not limited to) the Canadian Institutes of Health Research (CIHR), the Canada Research Chairs program, the Canada Foundation for Innovation (CFI), the International Development Research Centre (IDRC), the Canadian International Development Agency (CIDA) and Health Canada (HC). Likewise, the program of Ridde et al. received funding from IDRC (through its Research for Health Equity program), from GHRI (through its Africa Health Systems Initiative program), and from CIHR (through its New Investigators program). It is apparent, therefore, that long-term partnerships have the ability to supersede and outlast their current funding arrangements when they have a coherent and compelling motivation that both keeps them together during periods of financial uncertainty, and renders them fundable across a spectrum of donors.

## Interdisciplinary responses to complex issues

The majority of the research partnerships featured in this supplement are, or strive to be, interdisciplinary both in their composition and in their approach to problem solving. We see, for example, partnerships that join development economists with physicians (Haddad et al.), biostatisticians with nurses (Kipp et al.), and infectious disease biologists with occupational health professionals (Yassi et al.). We also see partnerships that embrace qualitative and quantitative researchers, researchers and decision-makers, established and junior researchers, and academics and activists. Adding yet another layer of complexity, most of the partnerships featured here involve members from far-flung geographic regions and different cultural backgrounds. Spiegel et al. sum up all of these dimensions, when they explain that “knowledge sharing has fundamentally taken place within the dynamic of difference…three or more cultures, half a dozen disciplines, distinct paradigms, (and) three languages…” [5]. If the partnerships described in this supplement are representative of the wider field of global health research, it is clear that an interdisciplinary perspective is indeed a core characteristic of global health research.

Interdisciplinary approaches are characterized by the engagement of researchers from different disciplines in understanding and engaging in all components of a study and in sharing their different viewpoints regarding results and interpretations [6]. Interdisciplinary approaches are therefore considered more likely to lead to learning that goes beyond “additive” learning [7] and more likely to produce solutions that will have traction in the messiness of the real world. This view is reflected in some of the cases of this collection. Haddad et al. explain a process whereby the strengths of the two team leaders became mutually complementary, and then were further enhanced by the addition of different disciplines to the team, such that “the project became a crucible of intense learning, sending a strong message to the Canadian team that the narrow boundaries of economics had to be transcended to understand social systems with diverse caste and religious identities” [4].

Why is global health research a practice that causes its participants to strive to break down silos on so many fronts? Upon close reading, these articles suggest that at least part of the motivation lies in matching the means to the problem and to the end. In other words, research teams must be interdisciplinary in order to process and tackle the complex nature of global health issues, and their necessarily multi-faceted solutions. This is an era in which the inter-related socio-economic determinants of health are recognized but imperfectly understood [8,9]. Indeed, global health has been described as a ‘composite’ field; one that comprises biological, clinical and social health and is complemented by other disciplines such as engineering and political science [10]. Per force, the problems faced by global health partnerships are profoundly complex. Now more than ever, global health research requires the bridging of traditional divisions between disciplines in order to innovatively protect and promote health for all people [10,11].

While the bridging of disciplines is a fine theoretical ideal, how possible *is* this in the everyday reality of global health research programs? Both the literature and the experiences of these teams suggest that there are common impediments as well as key facilitating factors. At least one impediment stems from the possibly incompatible core values of different epistemological traditions. The depth of difference between traditions can often be appreciated in the downstream difficulty of reconciling different research methodologies. Beliefs and values about what constitutes sound research are often grounded in epistemologies and expressed in methodological approaches [12,13]. As explained by Ridde et al.:

“The challenges involved in the partnership …were more of an interdisciplinary nature than about North-South differences…the focus was on complementarity of theoretical and methodological approaches. For example, anthropologists most often use a very inductive process in conducting their research, whereas researchers in evaluation and public health generally organize their data using an analytical framework.” [14]

It follows then that in addition to linguistic bilingualism, global health research partnerships often strive for “methodological bilingualism”; a bilingualism that requires a minimum competency from all team members in each of the research methods [15]. Otherwise, researchers from various traditions find themselves at worse talking mutually incomprehensible methodological languages; and at best, producing ‘parallel’ results that fail to be integrated.

## Participatory action research

The case studies in this collection present a variety of experiences with participatory action research. Participatory action research involves a commitment to both study a system and to collaborate with members of that system to bring about desired change [16]. It demands the active collaboration of all stakeholders, leading ideally to a blurring of traditional roles defining “researcher” and “researched” and to an equal partnership between researchers and community stakeholders [17]. Theoretically, participatory action research involves all potential users of the research in the formulation, conduct, and application of the research and the research occurs in phased cycles (problem diagnosis, action planning, taking action, evaluating the actions, incorporating lessons, repeat) [18,19].

The phased-cycle nature of participatory action research is demonstrated in a number of the case studies. Cole et al. describe their decade-long program of research as a series of “iterative cycles of mixed methods research around particular questions, actions relevant to stakeholders, new proposal formulation and implementation followed by evaluation of impacts” [20]. A number of the partnerships initially worked together on fairly straightforward epidemiological surveys; and gradually moved towards a participatory action research orientation. This is exemplified by Spiegel et al., who describe moving from research for inquiry’s sake to impact-oriented investigation while maintaining rigor in methods. Similarly, Deslisle et al. reflect on the evolution of their program of research, in which “progress is being made in the type of research, impacts and partnership” [21]. All of this suggests that an enhanced degree of maturity is important for successful participatory action research. Maturity (in terms of the relationship between the primary research collaborators, the relationship between the researchers and the community stakeholders, and a nuanced understanding of the setting) and a willingness to invest in a phased-cycle of action and reflection are factors that privilege the uptake and the likely success of participatory action research.

It can be inferred from the case studies of this collection that a commitment to the ideals of participatory action research is often difficult to apply. The very complex confluence of sociocultural factors that contributed to the existing health inequities will not instantly dissipate in the face of even the best-designed action research intervention. As Cole et al. explain,

“Our research-action process sought to address (the underlying causes of health inequities)… but was constrained by them. During EcoSalud II interventions, vertical approaches to community leadership excluded broader social participation and limited some community members’ access…” [20].

A look at the relevant literature reveals some healthy skepticism about another fundamental tenet of participatory action research: the requirement of equal partnerships between researchers and community stakeholders. It has been pointed out that many action research projects, described as participatory, actually use differing levels of collaboration at distinct stages of the research. For example, community stakeholders may be more involved in diagnosing the problem and in taking action, but less involved in the analysis and writing of the findings [22]. Some argue that “dragging” participants through all of the research process is unjustified, as long as said participants help define the research questions and then eventually help to interpret the findings [18]. When the concept of community stakeholders is widened to include not just a single geographically defined human settlement, but also other groups of people such as health practitioners and policy makers, the challenges to full and equal stakeholder participation become ever greater.

## Research with a policy or practice impact orientation

Scholarly publishing is recognized as a measure of excellence in research. Global health research is certainly no exception. However, our experience in global health research reinforced by the case studies presented herein indicates that publishing alone is not sufficient. Taking action on modifiable determinants that affect health and health equity, and converting new knowledge into improved policies and programs are fundamental components of global health research. That is why, throughout this collection, the reader will notice the authors continually referring to the impact orientation of their work. As expressed by Delisle et al.; “The global health field owes it to itself to not only generate new knowledge and information but also to contribute to a population’s well-being” [21].

Through their storytelling the authors demonstrate the difficult and often capricious nature of knowledge translation. As such, the authors confirm known wisdom about the non-formulaic nature of policymaking and the sheer volume of factors that can influence the uptake of knowledge gleaned from research [23-26]. While each case study offers program-specific introspection about the factors governing policy and practice influence, overall the collection illuminates no pattern or best practice. In the world of policy and practice change, “outright success in terms of achieving specific, hoped-for change is rare, and the work that does influence policy is often unique and rarely repeated…” [27].

In this collection, only a few of the case studies describe situations in which the research provoked a traceable influence on policy at the national level. In their case study, Delisle et al. point to the influence of their research on policy and on practice at a national level in two countries.

“We believe that, because of our work, at least partly, nutrition related chronic diseases (NRCD) are being taken into account in Benin’s 2007 – 2016 National Health Development Plan…(and)…the primary education department in Burkina Faso is now considering introducing school lunch and nutrition programs not only in rural schools but also in urban schools…” [21]

The authors’ reluctance to claim direct sole responsibility for these changes points to a reality which complicates the lives of global health researchers and their donors—the “attribution problem” [27,28]. The causes of change (or stasis) in policy and practice are difficult to predict ahead of time and are often just as challenging to isolate and identify after the fact. This seems particularly true of the highest levels of government, as suggested by Cole et al., who describe the effort that was required from multiple civil society actors in order to restrict the use of hazardous pesticides in Ecuador. Thus the majority of the case studies in this supplement concentrate on describing changes that occurred in practice, and often at a very local level. These local changes in practice are often more tangible and a causal link can be more accurately attributed to the given research program.

Some of the case studies in this collection describe research that resulted in little or no discernable change to date. The case study of Mumtaz et al., for example, underlines the limitations of research when it confronts the more intractable and intransigent aspects of human society. In this case, relevant - and emotionally powerful -knowledge about gender inequities was generated. However, policymakers were not ready to address the deep-rooted ramifications of this knowledge. Because knowledge translation was a stated key objective of the research, the researchers had developed direct and ongoing engagement with government policymakers, who in turn expressed appreciation of the research results and saw them as important contributions to knowledge gaps. Despite these promising acknowledgements, however, policymakers have been unable to use the research results because “it is difficult for them to address the deep-seated … inequalities” [29].

## Concluding comments

It is in keeping with the nature of modern times that the practice of global health research defies tidy definition, as it grows and morphs and adapts on a continuous basis. For this reason, rather than exploring competing definitions we have preferred in this essay to deepen our understanding of global health research by examining some of the core characteristics that link ten exemplary global health partnerships. The core characteristics that we have chosen to explore (long-term partnerships, interdisciplinary approaches, participatory action research, and impact orientation) are simply those that are illustrated most vividly across the ten case studies. This is not intended to provide an exhaustive list nor a conclusive characterization of the practice of global health research. Furthermore, a listing of these four as separate characteristics risks over-simplification, since each is interwoven with the others. For example, the phased-cycle nature of participatory action research often demands long timeframes and thus long-term partnerships. Likewise, a desire to influence policy and practice requires that health researchers also understand socio-political contexts, and thus adopt interdisciplinary approaches.

In this essay we have not elaborated on the commendable achievements of this collection of partnerships. On this point, the case studies speak most eloquently for themselves. In describing long-term research programs as opposed to short-term discrete research projects, each set of authors has showcased the value and the potential of global health research partnerships.

We remain curious as to the applicability of our conceptualization of global health and global health research’s core characteristics. Does this collection of case studies represent a uniquely Canadian ‘take’ on global health research? Given that each partnership featured here is a mix of Canadian and international colleagues, and given that the partnerships have been described as egalitarian and mutually respectful, it follows that these case studies can be said to represent a ‘global’ approach to global health research. That said, it is also true that the research featured here is funded at least partially from Canadian sources, and thus might reflect the programming principles, priorities and concerns of these donors. We remain open to the idea that other collections of global health case studies, compiled using different criteria, might yield different visions about what characterises the practice of global health research.

Semantics, definitions, and core characteristics aside: at essence, this collection is a spirit-lifting demonstration that many people are incapable of living ‘life as usual’ when this requires ignoring the social injustices suffered by others. Furthermore, this collection is a demonstration that people are capable of joining forces across cultures, disciplines and sectors to forge long-term commitments to programs of research and real-world impact.

## List of abbreviations used

GHRI: Global Health Research Initiative; HIV/AIDS: Human Immunodeficiency Virus / Acquired Immune Deficiency Syndrome; CIHR: Canadian Institutes of Health Research; CFI: Canada Foundation for Innovation; IDRC: International Development Research Centre; CIDA: Canadian International Development Agency; HC: Health Canada; NRCD: nutrition related chronic disease

## Competing interests

Zoë Boutilier and Ibrahim Daibes serve as Program Officer and Senior Program Specialist, respectively, for the Global Health Research Initiative. Erica Di Ruggiero serves on the steering committee of the Global Health Research Initiative. The Global Health Research Initiative supported the assembly and publication of this supplement. The views expressed in this introductory article are those of the authors alone and do not represent the views of the Global Health Research Initiative (GHRI), the International Development Centre (IDRC), nor the Canadian Institutes of Health Research (CIHR).
